# Out-of-Plane Dielectric Susceptibility of Graphene in Twistronic and Bernal Bilayers

**DOI:** 10.1021/acs.nanolett.1c02211

**Published:** 2021-07-23

**Authors:** Sergey Slizovskiy, Aitor Garcia-Ruiz, Alexey I. Berdyugin, Na Xin, Takashi Taniguchi, Kenji Watanabe, Andre K. Geim, Neil D. Drummond, Vladimir I. Fal’ko

**Affiliations:** † National Graphene Institute, University of Manchester, Booth St.E., M13 9PL Manchester, U.K.; ‡ Dept. of Physics & Astronomy, University of Manchester, Manchester M13 9PL, U.K.; § National Institute for Materials Science, 1-1 Namiki, Tsukuba 305-0044, Japan; ∥ Department of Physics, Lancaster University, Lancaster LA1 4YB, U.K.; ⊥ Henry Royce Institute for Advanced Materials, Manchester M13 9PL, U.K.

**Keywords:** graphene, dielectric susceptibility, gating, screening, bilayer graphene excitons, twisted bilayer graphene

## Abstract

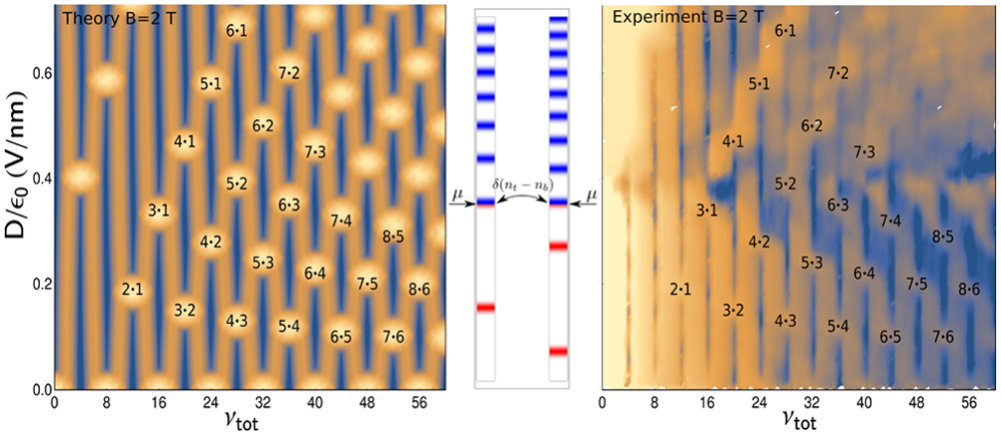

We describe how the out-of-plane dielectric polarizability of monolayer graphene influences the electrostatics of bilayer graphene—both Bernal (BLG) and twisted (tBLG). We compare the polarizability value computed using density functional theory with the output from previously published experimental data on the electrostatically controlled interlayer asymmetry potential in BLG and data on the on-layer density distribution in tBLG. We show that monolayers in tBLG are described well by polarizability α_*exp*_ = 10.8 Å^3^ and effective out-of-plane dielectric susceptibility ϵ_*z*_ = 2.5, including their on-layer electron density distribution at zero magnetic field and the interlayer Landau level pinning at quantizing magnetic fields.

Bilayer graphene^1−3^ is a two-dimensional (2D) material with electronic properties tunable over a broad range. The manifestations of the qualitative change of electronic characteristics of both Bernal (BLG) and twisted (tBLG) bilayer graphene, produced by electrostatic gating^3^ and interlayer misalignment,^4,5^ were observed in numerous experimental studies of the electronic transport in graphene-based field-effect transistor (FET) devices. These versatile electronic properties make FETs based on BLG and tBLG an attractive hardware platform for applications tailored^6−8^ for various quantum technologies. While, over the recent years, the fundamental electronic properties of bilayer graphene have been intensively studied, a mundane but practical characteristic of this material related to the out-of-plane dielectric susceptibility of graphene layers largely escaped attention of those investigations, despite several already recorded indications^9−13^ of its relevance for the quantitative modeling of the operation of graphene-based FET devices.

The out-of-plane dielectric susceptibility of a single graphene layer stems from the polarizability of its carbon orbitals, that is, from the mixing of π and σ bands by an electric field oriented perpendicular to the 2D crystal. Hence, we start by computing the out-of-plane polarizability of a graphene monolayer using *ab initio* density functional theory (DFT). We use that to estimate the effective dielectric susceptibility, ϵ_*z*_, of graphene and to design a recipe for implementing it in the self-consistent description of electrostatics in bilayers, both twisted and with Bernal stacking. For tBLG with twist angles outside the magic angle range,^5,8^ we perform a mesoscale analysis of the on-layer carrier densities, finding a good agreement with the earlier observations.^9−13^ Then, we implement the same recipe in the analysis of the interlayer Landau level pinning in strongly twisted bilayers. In this case, we also find an excellent agreement between the theoretical results and the measurements performed on a newly fabricated FET with a 30°-twisted tBLG. Finally, we take into account the out-of-plane dielectric susceptibility of a single graphene layer in the self-consistent analysis of the interlayer asymmetry gap in Bernal bilayer graphene,^1^ improving on the earlier calculations^3,14,15^ and successfully comparing the computed gap dependence on the vertical displacement field, Δ(*D*), with the earlier-measured interlayer exciton energies in gapped BLG.^16^

## Ab Initio Modeling of the Out-of-Plane Polarizability of Graphene

To determine the theoretical value of the out-of-plane dielectric polarizability of a graphene monolayer, we employ the CASTEP plane-wave-basis DFT code^17^ with ultrasoft pseudopotentials. We use a 53 × 53 × 1 *k*-point grid, a large plane-wave cutoff of 566 eV, and a variety of interlayer distances *c* along the *z*-axis to compute the total energy, , of graphene in a sawtooth potential, −*Dz*/ϵ_0_, centered on the carbon sites of the graphene layer (*D* being the displacement field and −*c*/2 < *z* < *c*/2). Then we determine (see additional note) the polarizability α in each cell of length *c* using the relation , with  being the vacuum energy. As the artificial periodicity, introduced in the DFT code, leads to a systematic error in the polarizability, *δα*(*c*) ∝ *c*^–1^, we fit the obtained DFT data with α(*c*) = α_*∞*_ + *a*/*c* + *b*/*c*^2^ and find α_*DFT*_ ≡ α(*c* → ∞) = 11 Å^3^ per unit cell of graphene with the Perdew–Burke–Ernzerhof (PBE) functional and α_*DFT*_ = 10.8 Å^3^ with the local density approximation (LDA).

These DFT values are close to the DFT-PBE polarizability reported in ref (18), α = 0.867 × 4π Å^3^ = 10.9 Å^3^, and when recalculated into an effective “electronic thickness” , where  is graphene’s unit cell area, we get 2.1 Å, comparable to the earlier-quoted “electronic thickness” of graphene.^12,20^ We also compared the computed DFT values with the polarizability computed using the variational (VMC) and diffusion (DMC) quantum Monte Carlo methods^21−26^ implemented in the CASINO code.^27^ In these calculations, we used the DFT-PBE orbitals generated using the CASTEP plane-wave DFT code^28^ and the orbitals being rerepresented in a localized B-spline “blip” basis. The localized basis improves the scaling of the quantum Monte Carlo (QMC) calculations and allows the use of aperiodic boundary conditions in the *z*-direction. The Jastrow correlation factor contained isotropic electron–electron, electron–nucleus, and electron–electron–nucleus terms as well as 2D plane-wave electron–electron terms,^29^ all optimized using VMC energy minimization.^30^ The DMC part of the calculations was executed with a time step of 0.01 Ha^–1^ and a target population of 4096 walkers. The resulting QMC out-of-plane polarizability of graphene is α ≈ 10.5 ± 0.2 Å^3^, which is also close to the above-quoted DFT-LDA value, so that, in the analysis below, we will use α_*DFT*_ = 10.8 Å^3^ for the polarizability of the graphene monolayer.

## Recipe for the Self-Consistent Analysis Electrostatics of Bilayers in the FET Configuration

**Figure 1:**
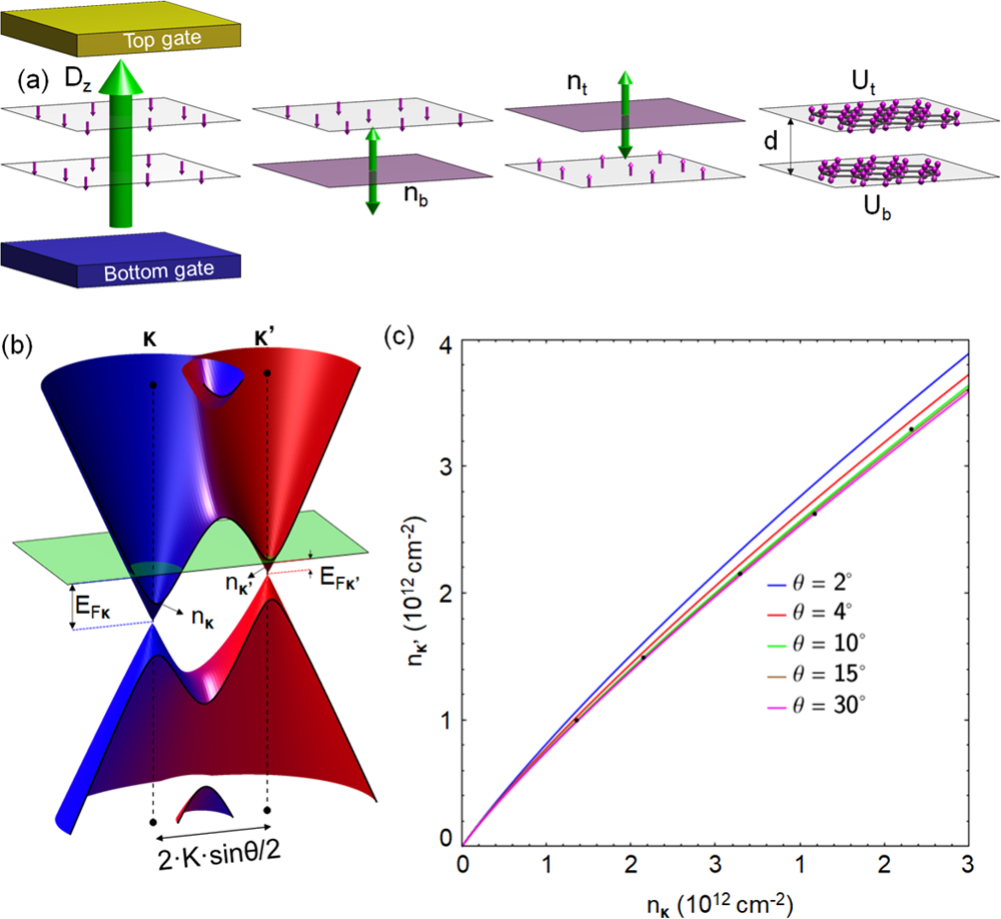
(a) Sketches illustrating how the dielectric polarizability of each monolayer enters in the electrostatics analysis of bilayers in eq 1. (b) Characteristic electron dispersion in tBLG (here, θ = 3°; *u* = 100 meV). Electron state amplitude on the top/bottom layer is shown by red/blue. (c) Minivalley carrier densities *n*_**κ**/**κ**′_ in a single-gated tBLG calculated for various misalignment angles outside the magic angle range, in comparison with the densities corresponding to SdHO measured^10^ in a tBLG flake with an unknown twist angle (black dots).

Now, we will use the microscopically computed polarizability α_*DFT*_ to describe the on-layer potentials and charges in bilayers, as a function of doping and vertical displacement field, *D*. For this, we note that *z*-polarization of carbon orbitals in each monolayer is decoupled from the charges hosted by its own π-bands because of mirror-symmetric charge and field distributions produced by the latter, see in Figure 1(a). Due to that, the difference between the on-layer potential energies, *u*, in the top and bottom layers of a bilayer, each with the electron density *n*_*b*/*t*_, has the form
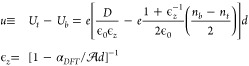
1Here, ϵ_*z*_ is the effective out-of-plane dielectric susceptibility, and *d* is the distance between the carbon planes in the bilayer. For the analysis below, we use *d* = 3.35 Å, resulting in ϵ_*z*_ = 2.6 for BLG, and *d* = 3.44 Å (as in turbostratic graphite^31^), leading to ϵ_*z*_ ≈ 2.5 for tBLG. This expression is applicable to the description of both BLG and tBLG in a FET, improving on the earlier-published studies^3,14,15^ where the out-of-plane dielectric susceptibility of graphene layers was missed out in the self-consistent band structure analysis.

## Electrostatics of tBLG – Mesoscale Modeling

To describe a twisted bilayer with an interlayer twist angle θ, we use the minimal tBLG Hamiltonian^4,5^
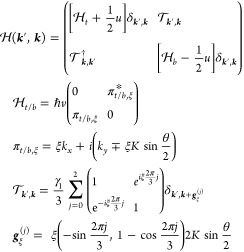
2Here, *v* = 10.2 · 10^6^ m/s is Dirac velocity in monolayer graphene. Eq 2 determines^4^ characteristic low-energy bands, illustrated in Figure 1(b) for 1 ≫ θ ≥ γ_1_/*ℏvK* ≡ 2° (away from the small magic angles ≤1°). This spectrum features two Dirac minivalleys at **κ** and **κ**′ (), which originate from the individual Dirac spectra of the monolayers. Each of those can be characterized by its own Fermi energy 
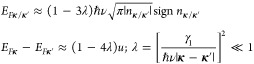
3and carrier density *n*_**κ**/**κ**′_, determined by the minivalley area encircled by the corresponding Fermi lines (as in Figure 1(b)). To mention, carrier densities *n*_**κ**/**κ**′_ can be determined experimentally from the 1/*B* period of Shubnikov–de Haas oscillations^9,10^ or by measuring the Fabry–Perot interference pattern in ballistic FET devices.^12^ The above expressions were obtained using linear expansion in small λ, taking into account that, due to the interlayer hybridization of electronic wave functions, the on-layer charge densities in eq 1 differ from the minivalley carrier densities, as
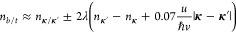
4

The latter feature makes the results of the self-consistent analysis of tBLG electrostatics slightly dependent on the twist angle, θ. We illustrate this weak dependence in Figure 1(c) by plotting the relation between the values of *n*_κ_ and *n*_κ′_ in a single-side-gated tBLG computed using eqs 3, 4, and 1 with ϵ_*z*_ = 2.5 and *d* = 3.44 Å. For completeness, on the same plot, we compare the computed *n*_**κ**_ and *n*_**κ**′_ values with the values recalculated from the periods of the earlier-measured SdHO^10^ in tBLG devices with an unknown twist angle. We find that our calculations closely reproduce those earlier-observed behavior for θ ≈ 10°, which correspond to the weak interlayer hybridization regime.

## Electrostatics of tBLG – Comparison with Experiments on a 30°-Twisted Bilayer

**Figure 2:**
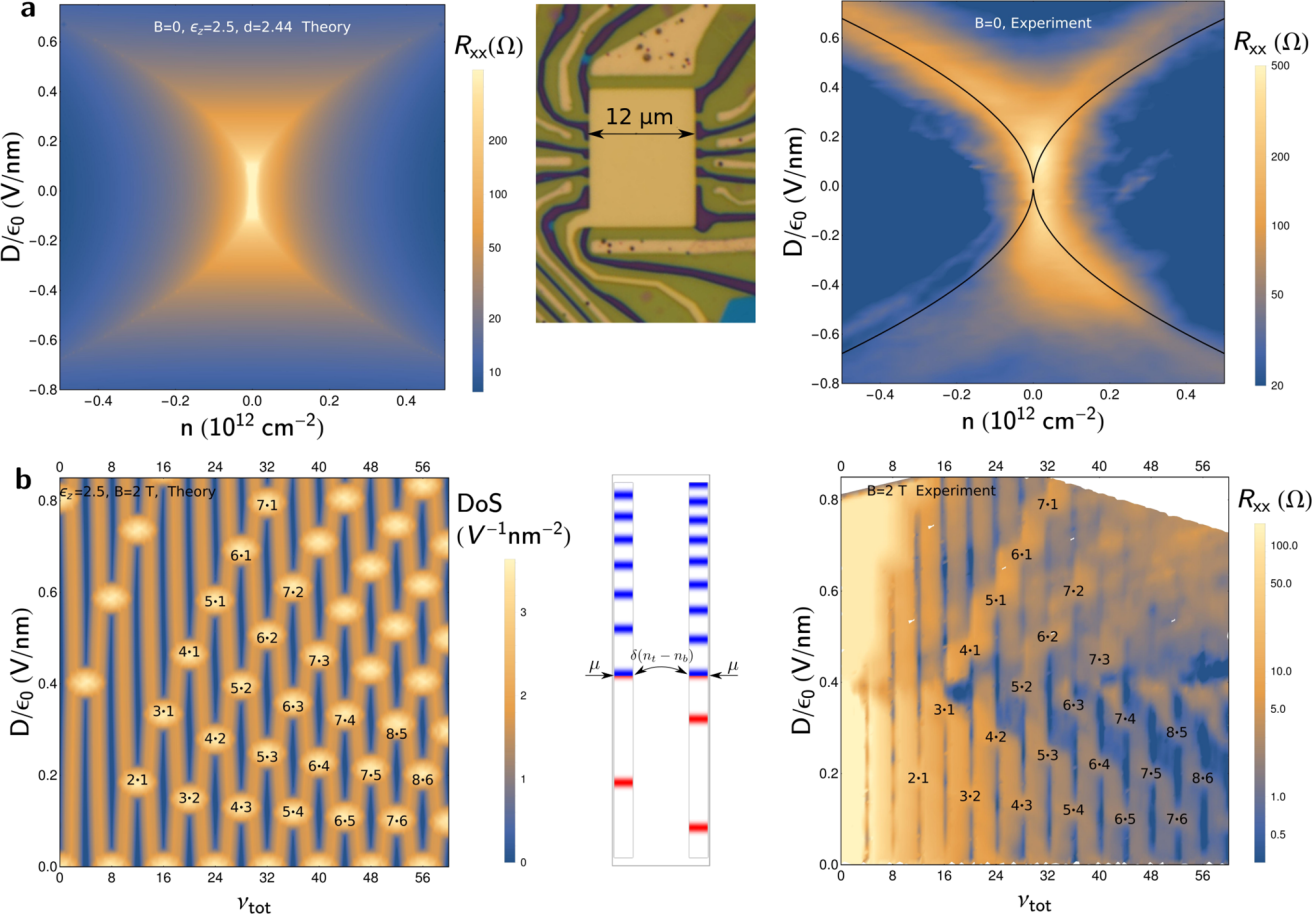
(a) Resistance map for a double-gated tBLG with a 30° twist angle, computed with ϵ_*z*_ = 2.5 and *d* = 3.44 Å (left) and measured (right) as a function of the total carrier density, *n*, and vertical displacement field, *D*, at *B* = 0 and *T* = 2 K. (b) Computed density of states of pinning LLs (left) and the measured resistance, ρ_*xx*_, (right) in a 30° tBLG at *B* = 2 T, plotted as a function of displacement field and filling factor. Bright regions correspond to the marked *N*_*t*_/*N*_*b*_ LL pinning conditions.

In fact, the weakest interlayer hybridization, λ → 0, appears in “maximally” misaligned layers in a tBLG with θ = 30°. In that case, the comparison between the theory an experiment is simplified by that *n*_*b*/*t*_ = *n*_**κ**/**κ**′_. Because of that, we fabricated a double-gated (top and bottom) multiterminal tBLG FET shown in the inset in Figure 2 and used it to measure the low-temperature (*T* = 2 K) tBLG resistivity at zero (*B* = 0) and quantizing magnetic field. In the experimentally studied device, tBLG was encapsulated between hBN films on the top and bottom, thus providing both a precise electrostatic control of tBLG for *B* = 0 measurements and its high mobility, enabling us to observe the quantum Hall effect at a magnetic field as low as *B* = 2 T. The measured displacement field and density dependence of resistivity is shown in the form of color maps on the right-hand side panels in Figure 2(a,b) for *B* = 0 and *B* = 2 T, respectively, where the form of “bright spots” of *R*_*xx*_ that appear in each of these two cases is affected by the interlayer charge transfer, controlled by tBLG electrostatics in eq 1.

For a quantitative comparison of the measured and modeled tBLG transport characteristics at *B* = 0, we assumed elastic scattering of carriers from residual Coulomb impurities in the encapsulating environment with a dielectric constant ϵ (ϵ ≈ 5 for hBN), with an areal density *n*_*c*_, screened jointly by the carriers in the top and bottom layers. The screening determines^32^ the Fourier form factor of the scatterers
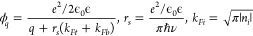
and the corresponding momentum relaxation rate of Dirac electrons^32^
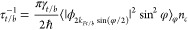
Then, in Figure 2(a), we compare the computed and measured tBLG resistivity. As in monolayer graphene,^32^ the density of states, γ_*t*/*b*_, cancels out from each , making the overall result, ρ_*xx*_ = ρ_*t*_ρ_*b*_/[ρ_*t*_ + ρ_*b*_], dependent on the carrier density only through the wavenumber transfer, 2*k*_*Ft*/*b*_ sin(φ/2), and screening. This produces ridge-like resistance maxima at *k*_*Ft*_ = 0 or *k*_*Fb*_ = 0, that is, when
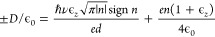
5

Lines corresponding to the above relation are laid over the experimentally measured resistivity map for a direct comparison.

For comparison between the theory and experiment at quantizing magnetic fields, we studied the Landau level pinning between the two graphene monolayers. In a magnetic field, graphene spectrum splits into Landau levels (LLs) with energies . In a twisted bilayer, infinite degeneracy of LLs gives a leeway to the interlayer charge transfer, which screens out the displacement field and pins partially filled top/bottom layer LLs, *N*_*t*_ and *N*_*b*_, to each other and to their common chemical potential, μ. As a result, we find that , as sketched in the inset in Figure 2(b). This LL pining effect also persists for slightly broadened (e.g., by disorder) LLs. Taking into account a small Gaussian LL broadening, Γ, we write,
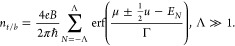
6Then, we solve self-consistently eq 1 and compute the total density of states (DoS) in the bilayer. The computed DoS for *B* = 2 T and Γ ≈ 0.5 meV is mapped in Figure 2(b) versus displacement field and the total tBLG filling factor, ν_*tot*_ = *hn*/*eB* (*n* = *n*_*t*_ + *n*_*b*_). Here, the “bright” high-DoS spots indicate the interlayer LL pinning conditions, whereas the “dark” low-DoS streaks mark conditions for incompressible states in a tBLG. We compare this DoS map with ρ_*xx*_(*D*, ν_*tot*_) measured in the quantum Hall effect regime (similar to the ones observed earlier^9,10^ in other tBLG devices), where the high values of *R*_*x*_*x* manifest mutual pinning of partially filled LLs, and the minima correspond to the incompressible states. To mention, the computed pattern broadly varies upon changing ϵ_*z*_, whereas the value of ϵ_*z*_ = 2.5 gives an excellent match between the computed and measured maps in Figure 2(b).

## Electrostatics of Bernal Bilayers

Finally, we analyze the electrostatically controlled asymmetry gap^1^ in BLG, taking into account out-of-plane polarizability of its constituent monolayers. In this case, we use eq 1 with , recalculated from polarizability α_*DFT*_ using *d* = 3.35 Å, and the BLG Hamiltonian^1^
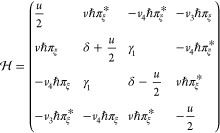
7which determines the dispersion and the sublattice (A/B) amplitudes, , in four (β = 1–4) spin- and valley-degenerate bands, . Here, π_ξ_ ≡ *ξk*_*x*_ + *ik*_*y*_, ***k*** = (*k*_*x*_, *k*_*y*_) is the electron wave vector in the valleys ***K***_ξ_ = ξ(4π/3*a*, 0), ξ = ±. The computed sublattice amplitudes, , determine the on-layer electron densities, which, in an undoped BLG with the Fermi level in the gap between bands β = 1, 2 and β = 3, 4, are
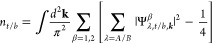
8

**Figure 3:**
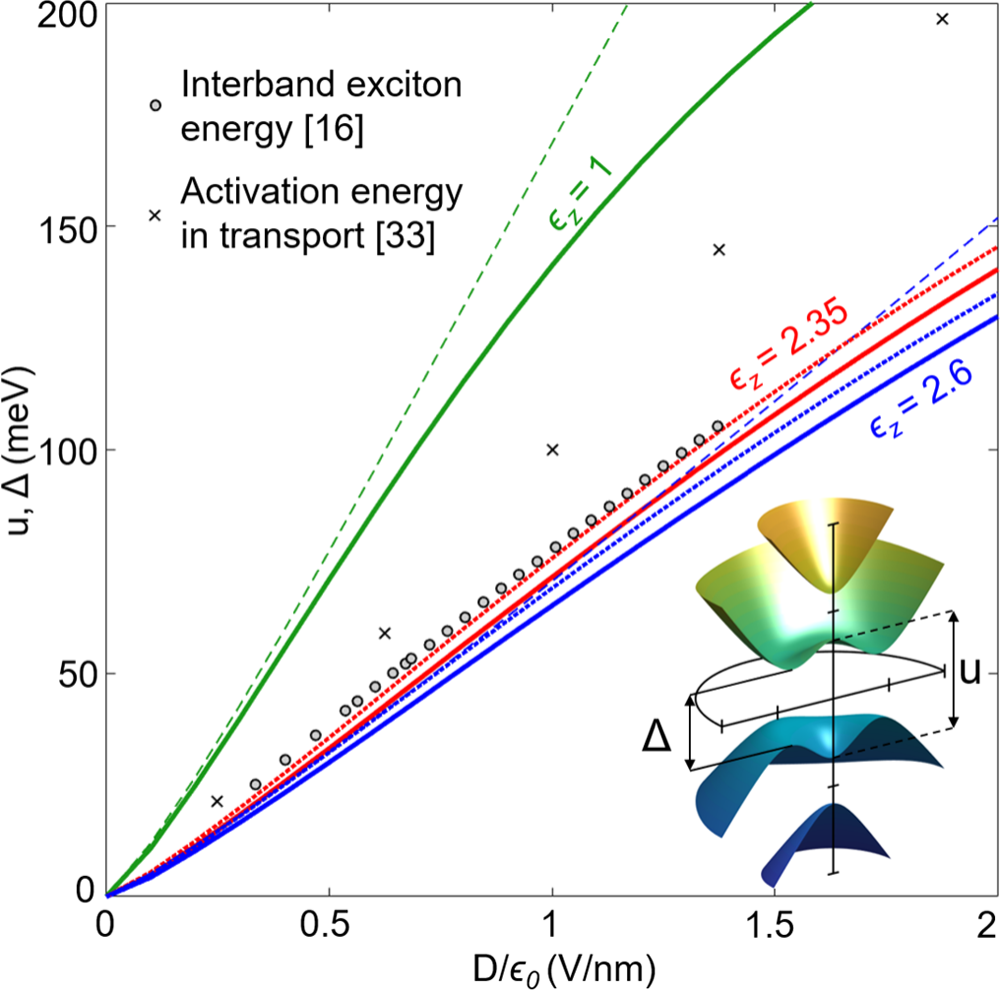
Interlayer asymmetry potential (dashed lines) and band gap (solid lines) in an undoped BLG, self-consistently computed with various values of ϵ_*z*_ = 1 (green), 2.6 (blue), and 2.35 (red) and compared to the optical gap measured in ref (16) (circles) and the transport gap^33^ (crosses). Here, we use^34,35^*v* = 10.2 · 10^6^ m/s, γ_1_ = 0.38 eV, *v*_3_ = 1.23 · 10^5^ m/s, *v*_4_ = 4.54 · 10^4^ m/s, δ = 22 meV, and *d* = 3.35 Å. Dotted lines show the values of the gap computed with γ_1_ = 0.35 eV and the same other parameters. The sketch illustrates four BLG bands (1,2 below and 3,4 above the gap) highlighting a small difference between *u* and Δ.

The on-layer potential energy difference, *u*, and a band gap, Δ, in the BLG spectrum (see inset in Figure 3), computed using self-consistent analysis of eqs 7, 8, and 1 with ϵ_*z*_ = 1 (as in refs (3), (14), and (15)) and with ϵ_*z*_ = 2.6, are plotted in Figure 3 versus displacement field, *D*. On the same plot, we show the values of lateral transport activation energy^36^ and the IR “optical gap”—interlayer exciton energy,^16^ measured earlier in various BLG devices. The difference between those two types of experimentally measured BLG gaps is due to that the single-electron “transport” gap is enhanced by the self-energy correction^37^ due to the electron–electron repulsion, as compared to the “electrostatic” value, *u*. In contrast, the interlayer exciton energy has a value close to the interlayer potential difference, *u*, because self-energy enhancement for electrons and holes is mostly canceled out by the binding energy of the exciton,^16,37^ an optically active electron–hole bound state. As one can see in Figure 3, *u* and Δ computed without taking into account a monolayer’s polarizability (ϵ_*z*_ = 1) largely overestimate their values. At the same time, the values of *u* and Δ obtained using ϵ_*z*_ = 2.6 appear to be less than the exciton energy measured in optics, for interlayer coupling across the whole range 0.35  < γ_1_ <  0.38 eV covered in the previous literature.^34,35,38−42^ This discrepancy may be related to that the interaction terms in the electron self-energy are only partially canceled by the exciton binding energy.^37^ It may also signal that the out-of-plane monolayer polarizability, α, is reduced by ∼10% when it is part of BLG, as the values of Δ computed with ϵ_*z*_ = 2.35 and γ_1_ = 0.35 eV agree very well with the measured optical gap values.

In summary, the reported analysis of the out-of-plane dielectric susceptibility of monolayer graphene shows that the latter plays an important role in determining the electrostatics of both Bernal and twisted bilayer graphene. We found that the DFT-computed polarizability of the monolayer, α = 10.8 Å^3^, accounts very well for all details of the electrostatics of twisted bilayers, including the on-layer electron density distribution at zero magnetic field and the interlayer Landau level pinning at quantizing magnetic fields. For practical applications in modeling of FET devices based on twisted bilayers, the polarizability of monolayer graphene can be converted to its effective dielectric susceptibility, ϵ_*z*_ ≈ 2.5, which should be used for the self-consistent electrostatic analysis of tBLG using eq 1 of this manuscript.

## Footnotes

Note that, at larger external fields, the energy abruptly becomes nonquadratic in *D* due to electronic density appearing in the artificial triangular well of the sawtooth potential, which sets the limits for the applicability of the DFT method we used. Also, we find that α is sensitive to the plane-wave cut-off energy at small external fields, which limits from below the range of *D* values we used in the analysis. We verified that the same polarizability results were obtained by directly evaluating the change in the dipole moment within the simulation cell when the external field is applied. Note that here we differ from some earlier studies of, e.g., bilayers,^18,19^ where the dielectric screening contribution has not been separated from the contribution resulting from charge redistribution across the layers.
